# Comparison of En Masse Repair versus Separate Double-Layer Repair for Delaminated Rotator Cuff Tears: A Systematic Review and Meta-Analysis

**DOI:** 10.3390/jcm13051393

**Published:** 2024-02-28

**Authors:** Kyun-Ho Shin, Il-Tae Jang, Seung-Beom Han

**Affiliations:** 1 Department of Orthopedic Surgery, Yeson Hospital, Bucheon 14555, Republic of Korea; 2 Department of Neurosurgery, Gangnam Nanoori Hospital, Seoul 06048, Republic of Korea; nanoori_research@naver.com; 3 Department of Orthopedic Surgery, Anam Hospital, College of Medicine, Korea University, Seoul 02841, Republic of Korea; oshan@korea.ac.kr

**Keywords:** arthroscopy, rotator cuff tear, delamination, separate layer, meta-analysis

## Abstract

Background: Delamination of cuff tendons has a negative impact on outcomes following arthroscopic rotator cuff repair (RCR). The purpose of this study is to compare en masse repair (EMR) and separate double-layer repair (SDLR) for delaminated rotator cuff tears. Methods: A systematic literature search was conducted on major databases (MEDLINE/PubMed, EMBASE, Cochrane Library, and Scopus) until 1 June 2023. Comparative studies with a minimum 24-month follow-up of patients undergoing arthroscopic RCR for delaminated tears were included. The outcomes assessed retear rates and functional outcomes. Results: Five eligible studies involving 325 cases were analyzed. The meta-analysis showed no significant difference in retear rates between SDLR and EMR for delaminated tears (OR = 0.73, 95% CI: 0.35–1.49). However, the meta-analysis demonstrated a significant intergroup difference in favor of the SDLR for the total Constant score (SMD = 0.68, 95% CI: 0.35 to 1.02), SST score (SMD = 0.37, 95% CI: 0.02 to 0.71), and postoperative range of abduction (SMD = 0.34, 95% CI: 0.03 to 0.64). Conclusion: The evidence suggests that the SDLR in arthroscopic RCR for delaminated rotator cuff tears leads to improved short-term functional outcomes and range of motion compared to EMR. However, there is no significant difference in retear risk between the two approaches.

## 1. Introduction

Rotator cuff tears are a common type of shoulder injury that causes shoulder pain and dysfunction, leading to limitations in daily activities [1]. Arthroscopic rotator cuff repair (RCR) has proven to be effective in restoring the anatomy of the original rotator cuff tendon insertion, improving shoulder strength and function, and reducing pain when conservative treatments have failed [2,3].

During the surgical procedure, the delamination of the posterosuperior cuff, which refers to a horizontal lesion between the superficial and deep fiber layers, is frequently observed [4,5]. The detection of cuff tendon delamination depends on the viewing portal utilized. While a posterior viewing portal only allows for the identification of 11% of delaminations, a lateral viewing portal enables visualization of 100% of the delaminated tendons [6]. The prevalence of delamination in the cuff tendon has been reported to range between 38% and 92% [6,7,8,9,10]. Moreover, posterosuperior cuff delamination is considered a negative prognostic factor for cuff healing and is associated with a high retear rate [6,10,11,12]. Therefore, it is of paramount importance to accurately identify and appropriately address cuff tendon delamination during surgical procedures.

The superior layer represents the rotator cuff tendon, while the inferior layer corresponds to the superior joint capsule [13,14]. En masse repair (EMR) is performed by passing a suture through both the bursal and articular layers simultaneously [15]. Although EMR has shown favorable results [7,15,16], individual repair of each layer, known as separate double-layer repair (SDLR), has been recommended to achieve anatomical restoration and prevent tension and length mismatch between the layers [9,17,18,19]. The debate centers around whether SDLR of delaminated rotator cuff tears can lower retear rates and improve outcomes [20,21,22,23]. A previous systematic review has addressed this issue [24]. However, the clinical evidence supporting SDLR in delaminated rotator cuff tears remains inconclusive, as the inadequate inclusion of patient cohorts comprising both delaminated and non-delaminated rotator cuff tears raises concerns about the reliability of the conclusions. Furthermore, several high-quality comparative studies have been published in recent years [25,26,27]. These additional studies can provide a better understanding of the effects of SDLR in delaminated rotator cuff tears.

The primary aim of this study is to conduct a meta-analysis and systematic review comparing the clinical outcomes and retear rates associated with EMR and SDLR techniques for delaminated rotator cuff tears. Our hypothesis is that SDLR will significantly improve retear rates and functional outcomes compared to EMR in delaminated rotator cuff tears. The findings of this study will help guide clinicians in making informed decisions regarding the optimal surgical technique for treating delaminated rotator cuff tears.

## 2. Materials and Methods

In this study, we followed the guidelines provided by the Preferred Reporting Items for Systematic Reviews and Meta-Analyses (PRISMA) to ensure the accuracy and transparency of our reporting [28]. This study is registered with the ResearchRegistry, and the unique identifying number is reviewregistry1765. The tasks involved in the study, such as study screening and selection, quality assessment, data extraction, and result pooling, were carried out independently by two authors. To maintain consistency and resolve any discrepancies, a third independent author reviewed the data and reached a consensus. The inter-reviewer process was evaluated for reliability using the kappa statistic (κ), which measures the level of agreement between the reviewers. The reliability values for all tasks ranged from 0.95 to 1.00, indicating a high level of agreement between the reviewers. This rigorous methodology enhances the reliability and validity of our study findings.

### 2.1. Inclusion and Exclusion Criteria

1. Participants: The study included patients who underwent primary arthroscopic RCR specifically for delaminated full-thickness rotator cuff tears.
2. Interventions: In the intervention group (SDLR group), the patients received arthroscopic RCR for the delaminated cuff tendon using the SDLR technique.
3. Comparisons: The control group (EMR group) underwent arthroscopic RCR for the delaminated rotator cuff tendon using the EMR technique.
4. Outcomes: The study assessed various outcomes, including rotator cuff retear rates (evaluated through magnetic resonance imaging or sonography) and functional outcomes measured by the Constant score, American Shoulder and Elbow Surgeons (ASES) score, University of California at Los Angeles (UCLA) score, Simple Shoulder Test (SST) score, visual analog scale (VAS), and postoperative range of motion (ROM).
5. Follow-up: The included studies required a minimum clinical follow-up of 24 months.
6. Study design: The study considered both randomized controlled trials (RCTs) and non-randomized case-control studies as eligible for inclusion.

During the first stage of screening, duplicated publications were removed, and two independent authors screened all the titles and abstracts. The full text of the articles was reviewed in the second stage of the screening process to select articles that met the inclusion and exclusion criteria.

### 2.2. Search Strategy

To identify pertinent articles comparing the outcomes associated with EMR and SDLR techniques in patients undergoing primary arthroscopic RCR for delaminated rotator cuff tears, a comprehensive search was conducted on 1 June 2023 across renowned databases including MEDLINE/PubMed, Cochrane Library, Embase, and Scopus. The search strategy employed a meticulous combination of keywords, Medical Subject Headings (MeSH) terms, and their respective variations within the [Title/Abstract] field of the search engines. The utilized terms comprised “rotator cuff”, “supraspinatus”, “infraspinatus”, “subscapularis”, “teres minor”, “rotator cuff” [MeSH term], “rotator cuff injuries” [MeSH term], “rotator cuff arthropathy” [MeSH term], in conjunction with “delamination” or “delaminated”. Language restriction was implemented during the search process, limiting the inclusion of studies conducted only in the English language. While the application of language restriction may introduce bias, it is widely accepted that the inclusion of English-language studies in systematic reviews does not significantly affect the overall information yielded [29].

Additionally, a thorough examination of the reference lists of the selected articles was conducted to ensure the inclusion of any potentially overlooked relevant studies.

### 2.3. Data Collection and Quality Assessment

Data extraction was performed by two independent authors based on the descriptive information obtained from the selected studies. The extracted data encompassed the following aspects: (1) study characteristics, such as the name of the first author, publication year, and country; (2) patient demographics, including the number of patients, sex, and age; (3) details regarding the size and characteristics of rotator cuff tendon tears; (4) surgical procedures, including the specific repair method employed and any concomitant procedures performed; (5) information regarding rehabilitation programs implemented; (6) duration of the follow-up period; and (7) outcomes of interest. The Robins-I tool [30] was used to assess controlled non-randomized before and after studies. The Risk of Bias (RoB) 2 tool [31] was applied for randomized trials. Visual plots representing the risk of bias were generated using Robvis [32].

### 2.4. Statistical Analysis

For continuous outcomes, the intergroup difference in mean outcomes divided by the standard deviation of the difference was calculated as the standardized mean difference (SMD) and presented along with a 95% confidence interval (CI). Dichotomous outcomes were analyzed using the odds ratio (OR) with a 95% CI. Meta-analyses were conducted to combine the effects and calculate the corresponding 95% CIs. The presence of statistical heterogeneity was assessed through a test of homogeneity based on the χ^2^ test, I^2^ statistics, and Q statistics. Given the observed methodological heterogeneity among the included studies, random-effects models (DerSimonian–Laird method) were utilized for meta-analyses. In cases where I^2^ was 50% or higher, indicating significant heterogeneity, a “leave-one-out” sensitivity analysis was conducted to identify the potential source of heterogeneity [33]. Publication bias was not assessed due to the small number of included studies (*n* < 10) [34]. All statistical analyses were conducted using Rstudio v.1.0.143 (RStudio Inc., Boston, MA, USA), with a significance level set at *p* < 0.05.

## 3. Results

### 3.1. Search Results

**Figure 1:**
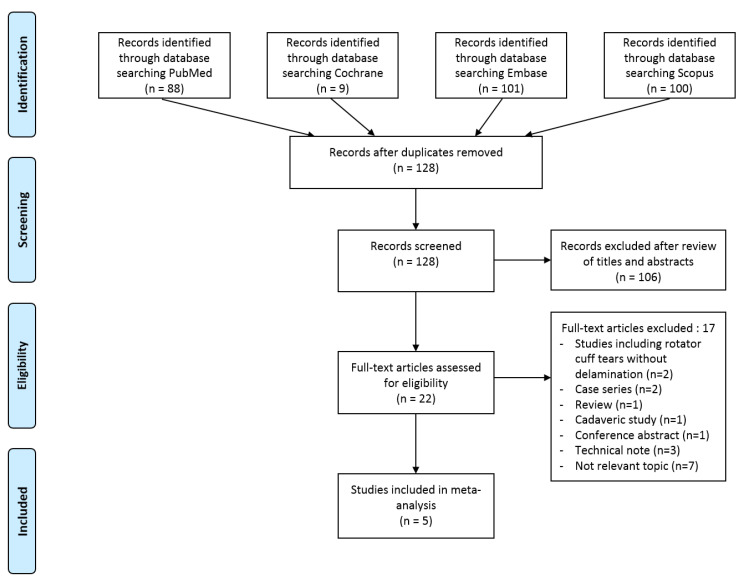
PRISMA flow diagram.

Figure 1 provides an overview of the process used to identify and select studies for inclusion. Initially, a total of 298 articles were identified through the literature search. After removing 170 duplicates, the remaining 128 articles underwent screening based on their titles and abstracts. Subsequently, 22 full-text articles were assessed for eligibility. Out of these, 17 articles were excluded as they did not meet the predetermined inclusion criteria. Ultimately, five articles were included in the meta-analysis [21,23,25,26,27].

### 3.2. Study Characteristics

Table 1 presents the baseline characteristics of the studies included in this meta-analysis. A total of 325 patients, comprising 154 patients who underwent SDLR and 171 patients who underwent EMR, were included. The mean age of the patients varied between 62.8 and 69.6 years, and the follow-up period ranged from 24 to 34.9 months. Four studies [21,23,25,27] enrolled patients with medium- to large-sized full-thickness rotator cuff tears, while one study conducted by Okubo et al. [26] included patients with large-sized or massive rotator cuff tears. The repair technique and postoperative rehabilitation program are summarized in Table 2.

### 3.3. Quality Assessment

**Figure 2:**
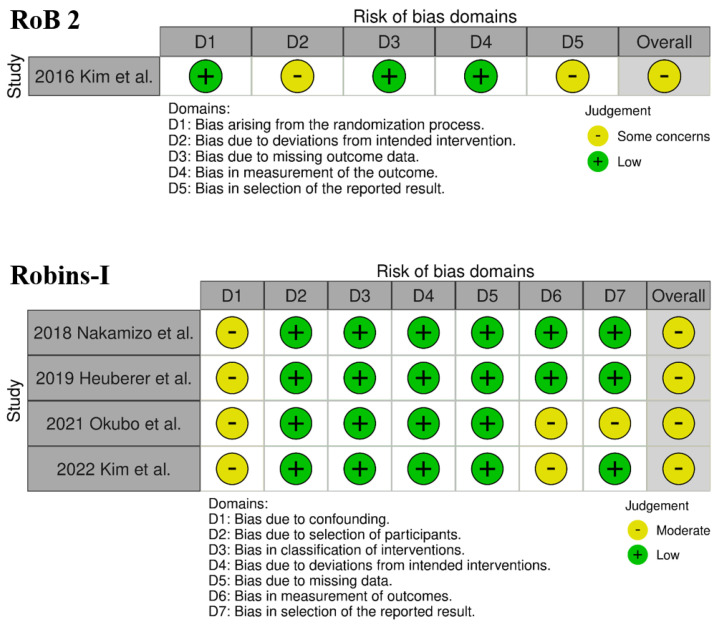
“Traffic light” plots of the quality assessment using RoB 2 and Robins-I.

**Figure 3:**
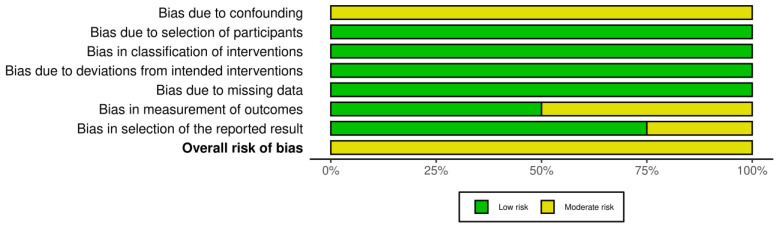
Summary plot of the distribution of risk-of-bias judgments in each bias domain of all outcomes assessed in studies judged with Robins-I.

The evaluation of the risk of bias in the included studies is depicted in Figure 2 and Figure 3.

One RCT conducted by Kim et al. [23] was assessed to have an unclear risk of reporting bias because of the absence of a pre-reported protocol. Overall, this study was categorized as having some concerns of bias. Concerning the four comparative studies, all of them were identified to carry a risk of time-varying confounding. Additionally, two studies [26,27] had an unclear risk of bias in outcome measurement, and one study by Okubo et al. [26] exhibited an unclear risk of bias in the selection of the reported result.

### 3.4. Meta-Analysis Results

#### 3.4.1. Retear

**Figure 4:**
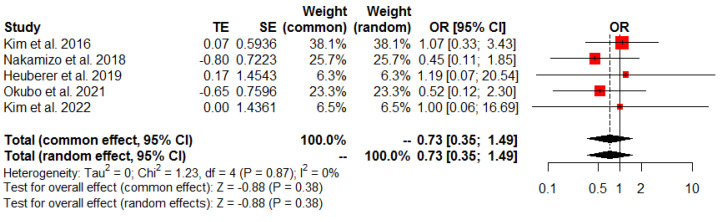
Postoperative overall retear rates.

The findings of the meta-analyses regarding retear rates are summarized in Table 3. All studies included in the analysis reported the retear rate, which was evaluated using postoperative MRI. The pooled results indicated that there was no significant difference in the postoperative retear rates between the SDLR and EMR groups (OR 0.73, 95% CI: 0.35 to 1.49, *p* = 0.38, I^2^ = 0%; Figure 4).

#### 3.4.2. Functional Outcomes

**Figure 5:**
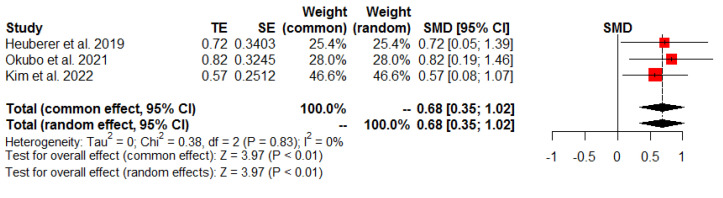
Postoperative total Constant score.

**Figure 6:**
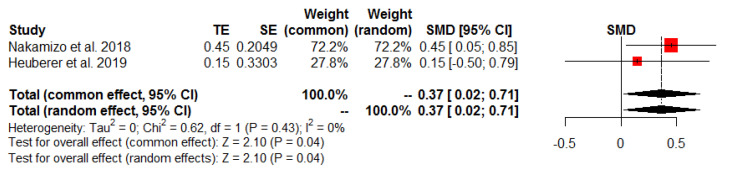
Postoperative Simple Shoulder Test.

The findings of the meta-analyses of functional outcomes are summarized in Table 3, while Table 4 presents the pre- and postoperative functional outcome scores from included studies. Three studies [25,26,27] assessed the total Constant score, while two studies [21,25] evaluated the SST score postoperatively. The meta-analysis revealed a significant intergroup difference in favor of the SDLR group for the total Constant score (SMD 0.68, 95% CI: 0.35 to 1.02, *p* < 0.01, I^2^ = 0%; Figure 5) and SST score (SMD 0.37, 95% CI: 0.02 to 0.71, *p* = 0.04, I^2^ = 0%; Figure 6).

Two studies [21,27] examined VAS and UCLA scores, while two studies [25,27] evaluated the ASES score postoperatively. The meta-analysis results indicated no significant intergroup difference in the postoperative VAS (SMD 0.14, 95% CI: −0.45 to 0.17, *p* = 0.37, I^2^ = 0%; Table 3), UCLA score (SMD 0.26, 95% CI: −0.05 to 0.56, *p* = 0.10, I^2^ = 35%; Table 3), and ASES scores (SMD 0.28, 95% CI: −0.11 to 0.66, *p* = 0.16, I^2^ = 0%; Table 3).

**Figure 7:**
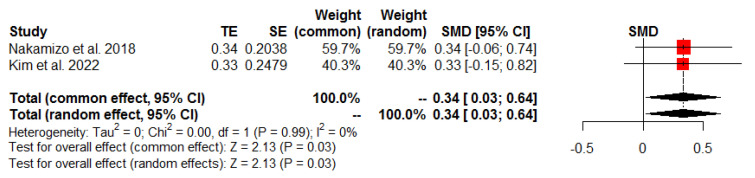
Postoperative range of abduction.

Three studies reported postoperative ROM, including forward flexion, abduction, and external rotation [21,26,27]. The pooled results showed a significant intergroup difference in favor of the SDLR group for the range of abduction (SMD 0.34, 95% CI: 0.03 to 0.64, *p* = 0.03, I^2^ = 0%; Figure 7). However, there was no significant intergroup difference in the range of forward flexion and external rotation (Table 3).

#### 3.4.3. Sensitivity Analyses

Significant heterogeneity was observed in the pooled results of the postoperative range of external rotation. Sensitivity analyses highlighted the study conducted by Nakamizo et al. as a potential source of this heterogeneity [21]. As a result, this study was excluded from the meta-analysis, as shown in Supplementary Figures S1 and S2.

## 4. Discussion

The key findings of our study suggest that the SDLR technique for delaminated rotator cuff tears demonstrated significantly better short-term functional outcomes in terms of the Constant score, SST scores, and ROM when compared to the EMR technique. However, there was no significant difference between the two groups in terms of retear rates.

Previous literature has consistently indicated that delamination of cuff tendons is more prevalent than initially expected and serves as a negative prognostic factor for the outcome of RCR [6,7,8,9,10,11,12,35]. The bursal layer corresponds to the rotator cuff tendon, whereas the articular layer corresponds to the superior joint capsule. Consequently, the articular capsular layer and bursal tendon layer exhibit distinct retraction directions, with the articular capsular layer exhibiting a tendency for more medial retraction [13,14].

Conventional EMR repair techniques, which involve piercing the delaminated rotator cuff tear with a single layer, often fail to achieve accurate anatomic restoration of the articular capsular layer and are susceptible to issues such as overtension or tension mismatch between the superior and inferior layers. The presence of varying strain levels across different tendon layers during shoulder motion has been established through both in vivo and in vitro experiments [5,36]. Consequently, it becomes nearly inevitable to encounter tension mismatch when employing the EMR technique for a retracted articular layer. Excessive tension or tension mismatch in the tendon has already been recognized as a problematic determinant affecting functional outcomes following RCR [37,38,39,40]. As a result, previous studies have reported unsatisfactory healing rates of cuff tendons in cases of delaminated rotator cuff tears treated with the EMR technique [15,35].

Biomechanical studies have emphasized the importance of the superior capsule in maintaining normal glenohumeral joint biomechanics, highlighting the significance of achieving accurate anatomical restoration of the superior capsular layer [19,41,42,43]. Similarly, in the context of delaminated cuff tear repair, it has been revealed that the anatomical reduction of the articular layer, specifically the superior capsule, plays a crucial role in determining clinical outcomes [44]. Therefore, several SDLR techniques that separately suture the superior capsular layer and the bursal tendon layer have been proposed to address the benefits of anatomically restoring the superior capsular layer and minimizing tension mismatch between the tendon layers [9,17,18,45,46,47].

Previous biomechanical studies have provided evidence that the anatomical reconstruction of the superior capsule and rotator cuff separately in cases of delaminated rotator cuff tears results in superior restoration of the footprint and higher peak load at failure. Moreover, these techniques have shown the ability to restore native biomechanics [19,48]. Although several clinical studies have been conducted comparing EMR and SDLR techniques for delaminated rotator cuff tears, the results have been inconsistent and inconclusive [20,21,22,23,25,26,27]. Therefore, the main objective of this study was to perform a meta-analysis to combine these findings and provide substantial evidence regarding the effectiveness of the SDLR technique. Our study findings support the notion that the SDLR technique leads to superior functional outcomes and improved ROM in the short-term follow-up compared to the EMR technique. However, there were no significant differences observed in terms of tendon integrity and the risk of retear between the two repair techniques.

Multiple factors contribute to the increased risk of retear following arthroscopic RCR. These factors encompass both demographic and preoperative characteristics, including advanced age, higher body mass index, diabetes, and fatty degeneration within the rotator cuff. Tear-related factors such as tear size, width, retraction, and the presence of a concomitant biceps lesion also play a significant role in retear risk [49,50]. However, it is worth noting that the extent of footprint coverage and the specific repair technique employed did not show a significant correlation with the risk of retear [49,50]. Despite the findings of Kim et al. [27], which suggested that the SDLR group exhibited a more homogeneous tendon signal and larger tendon width compared to the EMR group, the collective results of our meta-analysis did not show a significant difference in the risk of retear between these two specific repair techniques.

While the short-term follow-up results showed improved functional outcomes and range of motion with the use of the SDLR technique for delaminated rotator cuff tears, it is important to interpret these findings cautiously due to the absence of a significant difference in retear risk between the SDLR and EMR techniques, as well as the limited long-term data available. The SDLR technique, which involves independent reconstruction of both layers, can be challenging and time-consuming, requiring meticulous tendon release and separate suturing of each layer [6,10,51]. Therefore, the decision to use the SDLR technique in arthroscopic RCR for delaminated cuff tears should be carefully considered, taking into account the surgeon’s proficiency and the potential impact on the duration of the operation.

This review has several limitations that warrant acknowledgment. Firstly, the inclusion of a relatively low number of studies and participants, along with the incorporation of both randomized controlled trials (RCTs) and non-randomized comparative studies, should be considered. Although RCTs are generally considered the gold standard for causal inference, incorporating evidence from observational studies can strengthen the inference based solely on RCTs [52]. Therefore, this review included both RCTs and non-randomized comparative studies to provide more relevant and valid results for the research question. Secondly, considerable heterogeneity was observed among the included studies regarding tear size, repair techniques, rehabilitation protocols, and outcome assessment methods. This heterogeneity introduces variability into the analysis and should be taken into account when interpreting the findings. Thirdly, the potential risk factors and confounding variables that may influence postoperative outcomes, such as footprint preparation, smoking, body mass index, diabetes, and others, were not consistently documented or analyzed in the included studies. The lack of comprehensive consideration of these factors limits the ability to draw definitive conclusions about their impact on the outcomes. Fourthly, the analysis primarily focused on short-term outcomes due to the limited availability of long-term data. To establish a more definitive conclusion regarding the necessity of the SDLR technique in delaminated rotator cuff tears, future well-designed RCTs with larger sample sizes, longer-term follow-up, and adequate control of potential confounding factors are warranted. Conducting such studies would provide more robust evidence and enhance our understanding of the clinical implications of the SDLR technique.

## 5. Conclusions

In conclusion, the available evidence suggests that the SDLR technique in arthroscopic RCR for delaminated tears leads to improved short-term functional outcomes and range of motion compared to the EMR technique. However, there is no significant difference in retear risk between the two approaches. It is important to exercise caution when deciding to employ the SDLR technique, taking into account the surgeon’s expertise and potential implications for the duration of the operation.

## Figures and Tables

**Table 1:** Characteristics of included studies.

First Author (Year)	Study Design	Country	Level of Evidence	Sample Size (*n*)		Mean Age (Years)		Male *n* (%)		Follow Up		Type of Injury	Outcome Measurement
				Separate Double-Layer Repair	En Masse Repair	Separate Double-Layer Repair	En Masse Repair	Separate Double-Layer Repair	En Masse Repair	Separate Double-Layer Repair	En Masse Repair		
Kim et al. (2016) [23]	RCT	South Korea	II	34	48	65.5	65.2	11 (32.3)	16 (33.3)	Mean 25.9 months	Mean 25.8 months	Medium to large (tear size <5 cm) full-thickness supraspinatus tear with delamination	Retear rate at 12 months, VAS, ASES, SST, Constant, ROM
Nakamizo et al. (2018) [21]	Retrospective case-control study	Japan	III	46	52	64.1	65.8	20 (43.4)	28 (53.8)	Mean 27.6 months (minimum 24 months)	Mean 29.0 months (minimum 24 months)	Medium to large full-thickness supraspinatus and infraspinatus tear with delamination	Retear rate at 12 months, UCLA, SST, VAS, ROM
Heuberer et al. (2019) [25]	Prospective comparative study	Austria	III	17	20	62.8	64.8	5 (29.4)	10 (50)	12 and 24 months		Medium to large (2.0–3.5 cm) full-thickness supraspinatus and infraspinatus tear with delamination	Retear rate at 12 months, Constant, ASES, SST, VAS, ROM, SSV
Okubo et al. (2021) [26]	Retrospective case-control study	Japan	III	24	18	69.6	69.0	11 (45.8)	12 (66.7)	Mean 31.5 months (minimum 24 months)	Mean 34.9 months (minimum 24 months)	Large or massive rotator cuff tear with delamination	Retear rate, Constant, ROM
Kim et al. (2022) [27]	Retrospective case-control study	South Korea	III	33	33	63.9	64.7	14 (42.4)	13 (39.4)	Mean 29.1 months (minimum 24 months)		Medium to large (<5 cm) full-thickness supraspinatus tears with delamination	Retear rate at 12 months, VAS, ASES, Constant, UCLA, ROM

RCT, randomized controlled trial; VAS, visual analog scale; ASES, the American Shoulder and Elbow Surgeons score; SST, simple shoulder test; ROM, range of motion; UCLA, the University of California at Los Angeles score; SSV, the subjective shoulder value.

**Table 2:** Repair technique of rotator cuff tears and postoperative rehabilitation.

First Author (Year)	Repair Technique (SDLR Group)	Repair Technique (EMR Group)	Concomitant Procedures	Rehabilitation			
				Immobilization	Passive Motion Exercises	Active Motion Exercises	Strengthening Exercises
Kim et al. (2016) [23]	1 > Inverted mattress sutures configuration with medial knotless anchors 2 > Lateral anchors inserted into the lateral aspect of the footprint of the greater tuberosity for repair of the bursal layer	Suture bridge repair technique with medial row tension-free tying	Footprint preparation using burr until bleeding surface was exposed Acromioplasty for all type 2 and 3 acromions	Immobilization using a sling for 4 weeks postoperatively	From 4 weeks postoperatively for range-of-motion exercise program (passive, active-assisted, and active motion)		When passive shoulder range of motion was restored to 90%
Nakamizo et al. (2018) [21]	1 > Single row repair using a standard sliding knot and 2 half-hitches without cutting 2 > Medial sutures were passed through the bursal layer and fixed to the lateral row anchors using suture bridge repair technique without medial-row knot tying	Suture bridge repair technique without medial-row knot tying	Footprint preparation using shaver until bleeding surface was exposed Acromioplasty for a heel-type acromion	Immobilization using a sling for 4 to 6 weeks postoperatively	From 1 week postoperatively	From 4 to 6 weeks postoperatively after immobilization period	From 12 weeks postoperatively
Heuberer et al. (2019) [25]	1 > Articular layer repair by cinch loop configuration using medial anchors with looped sutures 2 > Medial sutures were passed through the bursal layer and fixed to the lateral row anchors using suture bridge repair technique without medial-row knot tying	Suture bridge repair technique with medial row knot tying	Footprint preparation using shaver and nanodrilling with a 1.4 mm K-wire Subacromial decompression and a tenodesis of the long head of the biceps were performed in every patient	Immobilization using a sling for 4 weeks postoperatively	Passive range of motion exercises were allowed immediately.	From 4 weeks postoperatively after immobilization period	From 12 weeks postoperatively
Okubo et al. (2021) [26]	1 > Single row repair using a standard sliding knot and 2 half-hitches without cutting 2 > Medial sutures were passed through the bursal layer and fixed to the lateral row anchors using suture bridge repair technique without medial-row knot tying	Suture bridge repair technique without medial-row knot tying	Subacromial bursectomy and subacromial osteophyte resection were performed	Immobilization using a sling for 3 to 4 weeks postoperatively	Passive range of motion exercises were allowed immediately	From 3 to 4 weeks postoperatively after immobilization period	From 12 weeks postoperatively
Kim et al. (2022) [27]	1 > Mattress sutures configuration with medial knotless anchors 2 > The “tip retention suture” of the medial knotless anchor was passed through the bursal layer and fixed to the lateral row anchors using suture bridge repair technique with medial-row knot tying	Suture bridge repair technique with medial-row knot tying	Footprint preparation using shaver and burr until bleeding surface was exposed Acromioplasty was performed if large and sharp subacromial bony spurs were seen after bursectomy Biceps tenotomy or tenodesis was performed based on the patient’s age and daily activities	Immobilization using a sling for 6 weeks postoperatively	From 4 weeks postoperatively	From 4–6 weeks postoperatively	From 12 weeks postoperatively

SDLR, separate double-layer repair; EMR, en masse repair.

**Table 3:** Summary of postoperative retear rates and functional outcomes between EMR and SDLR groups.

	OR or SMD	LL 95%CI	UL 95%CI	*p* Value	Number of Studies	Heterogeneity (%)	Analysis Model	Egger’s Test (*p*-Value)
Retear rate	0.73	0.35	1.49	0.38	5	0	Random	0.81
VAS	−0.14	−0.45	0.17	0.37	2	0	Random	NA
Constant score	0.68	0.35	1.02	<0.01	3	0	Random	0.32
SST score	0.37	0.02	0.71	0.04	2	0	Random	NA
ASES score	0.28	−0.11	0.66	0.16	2	0	Random	NA
UCLA score	0.24	−0.14	0.63	0.22	2	35	Random	NA
ROM (forward flexion)	0.09	−0.22	0.40	0.56	3	19	Random	0.60
ROM (abduction)	0.34	0.03	0.64	0.03	2	0	Random	NA
ROM (external rotation)	0.22	−0.16	0.60	0.26	2	0	Random	NA

EMR, en masse repair; SDLR, separate double-layer repair; OR, odds ratio; SMD, standardized mean difference; LL, lower limit; CI, confidence interval; UL, upper limit; NA, not applicable; VAS, visual analog scale; SST, simple shoulder test; ASES, the American Shoulder and Elbow Surgeons score; UCLA, the University of California at Los Angeles score; ROM, range of motion.

**Table 4:** Pre- and postoperative functional outcome scores from included studies.

First Author (Year)	Sample Size (*n*)		Preoperative Functional Outcomes		Postoperative Functional Outcomes		Follow Up	
	Separate Double-Layer Repair	En Masse Repair	Separate Double-Layer Repair	En Masse Repair	Separate Double-Layer Repair	En Masse Repair	Separate Double-Layer Repair	En Masse Repair
Kim et al. (2016) [23]	34	48	VAS; mean 5.7 ASES; mean 47.9 SST; mean 51.2 Constant score; mean 62.7	VAS; mean 6.5 ASES; mean 45.3 SST; mean 38.1 Constant score; mean 57.7	VAS; mean 1.2 ASES; mean 89.6 SST; mean 66.7 Constant score; mean 84.5	VAS; mean 2.0 ASES; mean 84.9 SST; mean 79.4 Constant score; mean 80.5	Mean 25.9 months	Mean 25.8 months
Nakamizo et al. (2018) [21]	46	52	VAS; 54.1 ± 19.4 SST; 5.1 ± 0.9 UCLA; 14.8 ± 2.2 Forward flexion; 145.6 ± 29.5 Abduction; 117.3 ± 36.4 External rotation; 43.6 ± 12.6 Internal rotation; L1	VAS; 52.6 ± 17.0 SST; 4.1 ± 1.9 UCLA; 13.7 ± 2.8 Forward flexion; 137.8 ± 38.8 Abduction; 112.0 ± 39.8 External rotation; 39.4 ± 14.5 Internal rotation; L2	VAS; 10.7 ± 11.9 SST; 10.0 ± 1.0 UCLA; 33.2 ± 2.3 Forward flexion; 165.8 ± 5.9 Abduction; 160.1 ± 9.1 External rotation; 53.7 ± 8.5 Internal rotation; T11	VAS; 13.4 ± 11.0 SST; 9.5 ± 1.2 UCLA; 32.0 ± 3.3 Forward flexion; 163.9 ± 6.0 Abduction; 154.8 ± 19.8 External rotation; 46.1 ± 9.4 Internal rotation; T12	Mean 27.6 months (minimum 24 months)	Mean 29.0 months (minimum 24 months)
Heuberer et al. (2019) [25]	17	20	VAS; 6 (range 2–10) ASES; 45.6 ± 15.1 SST; 5.1 ± 2.5 Constant; 49.1 ± 15.0 Forward flexion; 150 (range 60–170) Abduction; 150 (range 70–170) External rotation; 50 (range 30–80) SSV; 51.5 ± 20.7	VAS; 6.5 (range 2–10) ASES; 38.3 ± 18.4 SST; 4.2 ± 3.1 Constant; 42.9 ± 16.2 Forward flexion; 110 (range 10–170) Abduction; 140 (range 20–170) External rotation; 50 (range 5–90) SSV; 45.5 ± 17.0	VAS; 1 (range 0–4) ASES; 88.4 ± 11.2 SST; 9.4 ± 2.7 Constant; 78.8 ± 11.2 Forward flexion; 170 (range 90–180) Abduction; 160 (range 90–180) External rotation; 70 (range 60–90) SSV; 88.5 ± 14.8	VAS; 0 (range 0–5) ASES; 83.4 ± 16.5 SST; 9.0 ± 2.8 Constant; 68.8 ± 15.8 Forward flexion; 155 (range 110–180) Abduction; 152.5 (range 90–170) External rotation; 70 (range 50–80) SSV; 85.2 ± 11.7	12 and 24 months	
Okubo et al. (2021) [26]	24	18	Constant score; 45.5 ± 11.6 Forward flexion; 109 ± 30.1 External rotation; 47.2 ± 20.0 Internal rotation; L4.5	Constant score; 45.5 ± 14.3 Forward flexion; 105.6 ± 50.0 External rotation; 49.7 ± 16.8 Internal rotation; L4.8	Constant score; 87.6 ± 11.4 Forward flexion; 159.6 ± 19.7 External rotation; 71.4 ± 14.9 Internal rotation; L2	Constant score; 77.4 ± 13.6 Forward flexion; 158.9 ± 20.5 External rotation; 67.2 ± 13.7 Internal rotation; L2	Mean 31.5 months (minimum 24 months)	Mean 34.9 months (minimum 24 months)
Kim et al. (2022) [27]	33	33	VAS; 5.2 ± 2.0 ASES; 50.2 ± 19.9 UCLA; 17.6 ± 6.4 Constant; 62.4 ± 16.9 Forward flexion; 145.5 ± 43.3 Internal rotation; 3.3 ± 1.0	VAS; 5.3 ± 1.5 ASES; 49.1 ± 12.3 UCLA; 18.7 ± 5.0 Constant; 63.2 ± 12.3 Forward flexion; 152.1 ± 30.2 Internal rotation; 3.6 ± 1.3	VAS; 1.1 ± 0.9 ASES; 91.4 ± 6.8 UCLA; 31.2 ± 3.3 Constant; 91.4 ± 6.0 Forward flexion; 164.7 ± 7.5 Internal rotation; 3.7 ± 0.9	VAS; 1.1 ± 1.1 ASES; 88.3 ± 17.4 UCLA; 31.1 ± 6.0 Constant; 84.3 ± 16.4 Forward flexion; 165.9 ± 5.9 Internal rotation; 3.8 ± 0.8	Mean 29.1 months (minimum 24 months)	

The variables are presented as mean ± standard deviation or number (%). RCT, randomized controlled trial; VAS, visual analog scale; ASES, the American Shoulder and Elbow Surgeons score; SST, simple shoulder test; UCLA, the University of California at Los Angeles score; SSV, the subjective shoulder value.

## Data Availability

All data generated or analyzed during this study are included in this published article and its Supplementary Materials.

## Supplementary Materials

The following supporting information can be downloaded at https://www.mdpi.com/article/10.3390/jcm13051393/s1. Figure S1. Postoperative range of external rotation before sensitivity analyses. Figure S2. Sensitivity analysis for postoperative range of external rotation. Table S1. List of Evaluated Full Text Articles and Reasons for Exclusion.
