# Supplementary material for: Comparison of En Masse Repair versus Separate Double-Layer Repair for Delaminated Rotator Cuff Tears: A Systematic Review and Meta-Analysis
Source: J Clin Med. 2024 Feb 28;13(5):1393. doi: 10.3390/jcm13051393 (PMC10934360; doi:10.3390/jcm13051393)
Supplement: Supplementary file 1 [file jcm-13-01393-s001.zip › jcm-2833169-supplementary.pdf]

| Study | TE | SE | Weight (common) | Weight (random) | SMD [95% CI] |
|---|---|---|---|---|---|
| Nakamizo et al. 2018 | 0.85 | 0.2112 | 45.7% | 38.3% | 0.85 [ 0.43; 1.26] |
| Okubo et al. 2021 | 0.29 | 0.3134 | 20.8% | 27.5% | 0.29 [-0.32; 0.91] |
| Kim et al. 2022 | 0.18 | 0.2467 | 33.5% | 34.2% | 0.18 [-0.31; 0.66] |
| **Total (common effect, 95% CI)** | | | **100.0%** | **--** | **0.51 [ 0.23; 0.79]** |
| **Total (random effect, 95% CI)** | | | **--** | **100.0%** | **0.46 [ 0.02; 0.91]** |

Heterogeneity: $Tau^2 = 0.0915$; $Chi^2 = 4.85$, df = 2 (P = 0.09); $I^2 = 59\%$
Test for overall effect (common effect): Z = 3.54 (P < 0.01)
Test for overall effect (random effects): Z = 2.03 (P = 0.04)

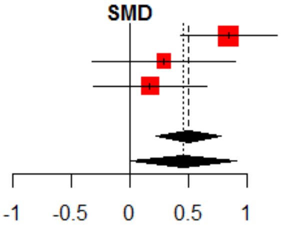


**Supplementary Figure 1.** Postoperative range of external rotation before sensitivity analyses

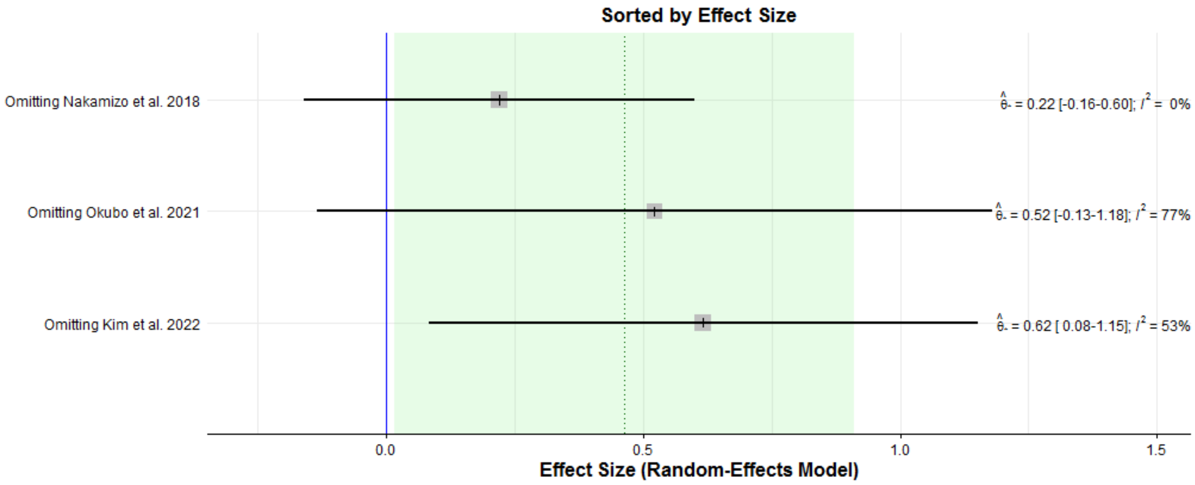


**Supplementary Figure 2.** Sensitivity analysis for postoperative range of external rotation

**Supplementary Table 1. List of Evaluated Full Text Articles and Reasons for Exclusion**

| Year | Journal | Author | Title | Reason for exclusion |
|---|---|---|---|---|
| 2014 | Arthroscopy techniques | Mori et al. | Arthroscopic lamina-specific double-row fixation for large delaminated rotator cuff tears | Technical note |
| 2015 | Journal of shoulder and elbow surgery | Gwak et al. | Delaminated rotator cuff tear: extension of delamination and cuff integrity after arthroscopic rotator cuff repair | Not relevant topic |
| 2016 | Journal of orthopaedic surgery and research | Cha et al. | Retraction pattern of delaminated rotator cuff tears: dual-layer rotator cuff repair | Innclusion of rotator cuff tears without delamination |
| 2016 | The American journal of sports medicine | Kim et al. | Surgical Results of Delaminated Rotator Cuff Repair Using Suture-Bridge Technique With All-Layers or Bursal Layer-Only Repair | Not relevant topic (comparison with bursal layer-only repair due to retraction) |
| 2016 | The American journal of sports medicine | Kim et al. | Conventional En Masse Repair Versus Separate Double-Layer Double-Row Repair for the Treatment of Delaminated Rotator Cuff Tears | Selected article |
| 2016 | Arthroscopy techniques | Mochizuki et al. | Repair of Rotator Cuff Tear With Delamination: Independent Repairs of the Infraspinatus and Articular Capsule | Technical note |
| 2017 | Arthroscopy: the journal of arthroscopic & related surgery | Kim et al. | Which is better between conventional en masse repair versus separate double-layer double-row repair for the treatment of delaminated rotator cuff tears: a prospective randomized study | Conference abstract |
| 2018 | BMC musculoskeletal disorders | Kakoi et al. | Clinical outcomes of arthroscopic rotator cuff repair: a retrospective comparison of double-layer, double-row and suture bridge methods | Study including rotator cuff tears without delamination |
| 2018 | Arthroscopy techniques | Heuberer et al. | An Arthroscopic Knotless Technique for Anatomical Restoration of the Rotator Cuff and Superior Capsule: The Double-Layer Cinch Bridge | Technical note |
| 2018 | Arthroscopy: the journal of arthroscopic & related surgery | Nakamizo et al. | Comparison of En Masse Versus Dual-Layer Suture Bridge Procedures for Delaminated Rotator Cuff Tears | Selected article |
| 2018 | Arthroscopy: the journal of arthroscopic & related surgery | Opsomer et al. | Arthroscopic Double-Layer Lasso Loop Technique to Repair Delaminated Rotator Cuff Tears | Case series |
| 2018 | Clinics in shoulder and elbow | Park et al. | Comparison of Clinical and Anatomical Outcomes between Delaminated Rotator Cuff Tear and Single Layer Rotator Cuff Tear | Not relevant topic |
| 2018 | The American journal of sports medicine | Pauzenberger et al. | Double-Layer Rotator Cuff Repair: Anatomic Reconstruction of the Superior Capsule and Rotator Cuff Improves Biomechanical Properties in Repairs of Delaminated Rotator Cuff Tears | Cadaveric study |

| | | | | |
|---|---|---|---|---|
| 2019 | Knee surgery, sports traumatology, arthroscopy | Heuberer et al. | The knotless cinch-bridge technique for delaminated rotator cuff tears leads to a high healing rate and a more favorable short-term clinical outcome than suture-bridge repair | Selected article |
| 2019 | Arthroscopy: the journal of arthroscopic & related surgery | Kim et al. | Morphologic Factors Related to Repair Outcomes for Delaminated Rotator Cuff Tears: A Minimum 2-Year Retrospective Comparison Study | Not relevant topic |
| 2019 | Orthopaedic journal of sports medicine | Mori et al. | Clinical and Radiographic Outcomes After Arthroscopic Lamina-Specific Double-Row Repair of Large Delaminated Rotator Cuff Tears in Active Patients | Case series |
| 2019 | Indian journal of orthopaedics | Pandey et al. | Clinical and Structural Outcomes after Arthroscopic Repair of Medium- to Massive-Sized Delaminated and Nondelaminated Rotator Cuff Tears | Not relevant topic |
| 2020 | Journal of orthopaedic surgery and research | Chen et al. | Separate double-layer repair versus en masse repair for delaminated rotator cuff tears: a systematic review and meta-analysis | Review |
| 2020 | The American journal of sports medicine | Heuberer et al. | Delaminated Rotator Cuff Tears Showed Lower Short-term Retear Rates After Arthroscopic Double-Layer Repair Versus Bursal Layer-Only Repair: A Randomized Controlled Trial | Not relevant topic (comparison with bursal layer-only repair) |
| 2021 | Orthopaedic journal of sports medicine | Kim et al. | Surgical Treatment Outcomes for Everted Bursal Flap of Delaminated Supraspinatus Tear | Not relevant topic |
| 2021 | Journal of the Société Internationale de Chirurgie Orthopédique et de Traumatologie | Okubo et al. | Comparison of three suture-bridge techniques for large or massive rotator cuff tear with delamination | Selected article |
| 2022 | The American journal of sports medicine | Kim et al. | A Propensity Score-Matched Comparison Between Knotless Layer-by-Layer and En Masse Suture Bridge Techniques for Delaminated Rotator Cuff Tears | Selected article |

**Search strategy (MEDLINE/PubMed)**

1. Rotator cuff [tiab] OR supraspinatus [tiab] OR infraspinatus [tiab] OR subscapularis [tiab] OR “teres minor” [tiab] OR “rotator cuff” [Mesh] OR “rotator cuff injuries” [Mesh] OR “rotator cuff tear arthropathy” [Mesh]
2. delamination [tiab] OR delaminated [tiab]
3. 1. AND 2.
